# Hypoxia‐induced proliferation of HeLa cells depends on epidermal growth factor receptor‐mediated arginase II induction

**DOI:** 10.14814/phy2.13175

**Published:** 2017-03-22

**Authors:** Bhuvana A. Setty, Natasha Pillay Smiley, Caitlyn M. Pool, Yi Jin, Yusen Liu, Leif D. Nelin

**Affiliations:** ^1^ Pulmonary Hypertension GroupCenter for Perinatal ResearchThe Research Institute at Nationwide Children's HospitalColumbusOhio; ^2^ Department of PediatricsThe Ohio State UniversityColumbusOhio

**Keywords:** Cancer, cell signaling, epidermal growth factor

## Abstract

Solid tumors can often be hypoxic in regions, and cancer cells can respond to hypoxia with an increase in proliferation and a decrease in apoptosis, leading to a net increase in viable cell numbers. We have recently found that in an osteosarcoma cell line, hypoxia‐induced proliferation depends on arginase II induction. Epidermal growth factor receptor (EGFR) has been shown to mediate the hypoxia‐induced cellular proliferation in some cancer cell lines. We hypothesized that hypoxia‐induced proliferation of HeLa cells would depend on arginase II induction and that this induction of arginase II would occur through EGFR activation. Exposure of HeLa cells to hypoxia resulted in an upregulation of arginase II mRNA and protein levels, with no effect on arginase I expression. Hypoxia also resulted in significantly greater viable cell numbers than did normoxia. The hypoxia‐induced increase in viable cell numbers was prevented by either a small molecule inhibitor of arginase or siRNA targeting arginase II. Overexpression of arginase II resulted in an increase in viable cell numbers both in normoxia and hypoxia. Hypoxia caused a substantial induction of both epidermal growth factor (EGF) and EGFR. Preventing hypoxia‐induced EGFR expression using siRNA abolished hypoxia‐induced arginase II expression and the increase in viable cell numbers. Treatment with EGF in normoxia not only induced arginase II expression but also resulted in an increase in viable cell numbers. Blocking EGF interactions with EGFR using either an EGF neutralizing antibody or an EGFR antibody prevented the hypoxia‐induced increase in viable cell numbers. These results demonstrate an EGF/EGFR/arginase II pathway that is necessary for hypoxic proliferation in HeLa cells.

## Introduction

In studies on neoplastic cells, hypoxia promotes tumor growth rather than apoptosis (Iyer et al. 1999), perhaps explaining why hypoxia is associated with a more aggressive phenotype in neoplastic cells and a worse prognosis (Hockel et al. 1999). Despite hyper‐vascularization in solid tumors, hypoxic conditions in solid tumors result from an imbalance between the rates of tumor cell proliferation, new endothelial cell formation, and disorganized vascular supply (Vaupel and Mayer 2007).

L‐arginine is the substrate for arginase, of which two isoforms have been described, arginase I and arginase II (Jenkinson et al. 1996). Arginase I is a cytosolic enzyme that is highly expressed in the liver, while arginase II is a mitochondrial enzyme that is not expressed in the liver (Durante et al. 2007). The metabolism of l‐arginine by arginase leads to the production of L‐ornithine and urea. L‐ornithine can then be further metabolized to polyamines and proline, which are critical for cell proliferation, differentiation, and tissue repair (Li et al. 2001). Low arginine levels in vitro and in vivo arrest the growth of normal cells, and can selectively destroy malignant cells (Scott et al. 2000). This was first demonstrated in the 1950s in studies demonstrating that adding pharmacological doses of arginase to the media, which would substantially decrease media arginine levels, resulting in essentially arginine‐free media, in cultured fibroblasts and Jensen sarcoma cells inhibited mitosis (Wheatley et al. 2003). These results were subsequently demonstrated in rat liver extracts and other cell types (Holley 1967; Wheatley et al. 2003). These early studies demonstrated that when arginine was not available to cells and thereby not available to cellular arginase, cell growth was inhibited. Hypoxia has been shown to upregulate cellular arginase I and/or arginase II activity, although the response of arginase isoforms to hypoxia seems to be cell type‐specific (Pohjanpelto and Hölttä 1983). We have previously shown that hypoxia induces the expression of arginase II in human pulmonary microvascular endothelial cells (hPMVEC) and human pulmonary arterial smooth muscle cells (Chen et al. 2009; Toby et al. 2010). We have also reported that in human pulmonary microvascular endothelial cells hypoxia‐induced arginase II expression depended on epidermal growth factor receptor (EGFR) activation (Toby et al. 2010). EGFR has been shown to be activated by hypoxia in a number of cell types (Clarkson et al. 2005; Toby et al. 2010; Wouters et al. 2013). When EGFR is activated by binding of its preferred ligand, epidermal growth factor (EGF), two EGFR moieties form a homodimer and this causes *trans*‐phosphorylation and enables interactions with signaling molecules. In some cancers increased levels of circulating EGFR ligands have been found, and in these cancers preventing EGFR activation by inhibiting ligand‐binding has been shown to decrease tumor size (Herrera et al. 2008). However, the effect of a hypoxic environment on arginase expression has not been studied in cancer cells, nor has the role of EGFR signaling in hypoxia‐induced arginase expression. We hypothesized that hypoxia would increase the expression of arginase protein and mRNA in a human cervical cancer cell line, and that cellular proliferation would be inhibited by pharmacological inhibitors of arginase. We further hypothesized that hypoxia would result in EGF‐mediated EGFR activation, which is necessary for arginase II induction and proliferation.

We determined levels of arginase I and II expression in HeLa cells incubated in either normoxia or hypoxia. The role of arginase in the proliferation of hypoxic cells was assessed using small molecule inhibitors of arginase or siRNA targeting arginase II to determine the effects on viable cell numbers. We overexpressed arginase II to determine the effects on viable cell numbers. We measured EGF and EGFR expression in normoxia and hypoxia. We also used EGFR siRNA and a small molecule inhibitor of EGFR (AG1478) to determine the role of EGFR activation in arginase II expression and cellular proliferation. We utilized antibodies against EGF or EGFR to determine the role of EGF interactions with EGFR in hypoxia‐induced cell proliferation.

## Methods

### Cell Culture

HeLa cells were obtained from ATCC (Manassas, VA). The cells were cultured in Dulbecco's Modified Eagle Medium (DMEM) containing 10% fetal bovine serum (Mediatech Inc., Manassas, VA). The cells were grown to approximately 80–90% confluence and washed with PBS. Ten milliliters of fresh DMEM was placed on cells and incubated at 37°C in either 5% CO_2_, balance air (normoxia) or 5% CO_2_, 1% O_2_, balance N_2_ (hypoxia) for 24 h.

### siRNA silencing

siRNA as a mixture of four different siRNA against the target of interest was obtained against human arginase II (Silencer^®^ Select ID s1571, catalog number 4390824) or human EGFR (Silencer^®^ Select ID number s563, catalog number 4390824) as well as the appropriate scramble siRNA as a control from Invitrogen (Waltham, MA). HeLa cells were transfected with the appropriate siRNA using transfection reagents provided by the manufacturer as previously described (Chen et al. 2009; Toby et al. 2010; Setty et al. 2016). Cells were allowed to recover in the incubator at 37°C (5% CO_2_, 20% O_2_). After 24 h, cells were moved to hypoxia (5% CO_2_, 1% O_2_) for 24 h. After the incubation period, the cells were lysed for protein extraction. Other siRNA‐treated cells were plated in 6‐well plates after recovery, incubated in hypoxia for 48 h, and used in the proliferation studies described below.

### Protein isolation

Cells were washed twice with ice‐cold HEPES buffer and 200 *μ*L lysis buffer (0.2 mol/L NaOH, 0.2% SDS with the following added to each mL 30 min before use: 1 *μ*g aprotinin, 1 *μ*g leupeptin, and 1 *μ*g phenylmethylsulfonyl fluoride; all from Sigma, St Louis, MO) were placed on the cells. The plates were scraped, pipetted into sterile centrifuge tubes and placed on ice for 30 min. The cell lysates were centrifuged at 12,000*g* for 10 min. The supernatant was stored in 1.5 mL tubes at −80°C. Total protein concentration was determined by the Bradford method (BioRad, Hercules, CA).

### RNA isolation and real‐time PCR

Real‐time PCR for arginase I, arginase II, EGFR, and EGF were done as previously described (Chen et al. 2009; Toby et al. 2010; Setty et al. 2016). Briefly, RNA was isolated from cells using Trizol (Invitrogen, Carlsbad, CA). DNase treatment was performed on all samples using RNase‐free DNase (Super Array, SA Biosciences, Frederick, MD) followed by reverse transcription (Promega Corp., Madison,WI) and then analysis of cDNA by real‐time PCR using SYBR Green jumpstart Taq (Sigma). Primers were ordered from Invitrogen using the following sequences for human arginase I forward primer: 5′ TTGGCAATTGGAAG‐CATCTCTGGC 3′; reverse primers: 5′ TCCACTTGTGGTTGTCAGTGGAGT 3′. Human arginase II was amplified using the forward primer: 5′ TTAGCAGAGCTGTGT‐CAGATGGCT 3′ and the reverse primer: 5′ GGGCATCAACCCA‐GACAACACAAA 3′. Human EGFR‐forward primer: 5′ TTTGCTGATTCAGGCTTGG 3′; reverse primer: 5′ AGAAAACTGACCATGTTGCTTG 3′. Human EGF‐forward primer: 5′ GGGAATGGTTTATGCCCTAGAT 3′; reverse primer: 5′ CGCTGGGAACCATCCATATT 3′. 18S was amplified using the forward primer 5′ CCAGAGCGAAAGCATTTGCCAAGA 3′ and the reverse primer 5′ TCGGCATCGTTTATGGTCGGAACT 3′. For each reaction, negative controls containing reaction mixture and primers without cDNA were performed to verify that primers and reaction mixtures were free of template contamination. Relative arginase I, arginase II, EGFR, or EGF amounts were normalized to 18S expression using the ΔΔCT method (Livak and Schmittgen 2001). All samples were analyzed in duplicate. Data are shown as fold‐change relative to normoxia‐exposed control cells at each respective time point.

### Western blot analysis

The cell lysates were assayed for levels of arginase I, arginase II, EGFR protein, or phosphorylated EGFR using immunoblot analysis as previously described (Chen et al. 2009; Toby et al. 2010; Setty et al. 2016). Aliquots of cell lysate were diluted with 10 *μ*L SDS sample buffer, 4 *μ*L reducing agent, and appropriate amounts of deionized water. The samples were then heated to 80°C for 10 min, and then separated using SDS‐PAGE gel electrophoresis. The proteins were transferred to PVDF membranes, and blocked in TBS with 0.1% Tween (TBS‐T) containing 5% skim milk for 1 h, and then washed four times with TBS‐T and incubated at 4°C overnight with primary antibody against arginase I or arginase II (both 1:500; arginase I catalog number sc‐166920 and arginase II catalog number sc‐271443 from Santa Cruz Biotechnology, Santa Cruz, CA), EGFR (1:1000; catalog number ab131498, Abcam, Cambridge, MA), or pY845EGFR (1:1000; Antibody #2231 Cell Signaling, Danvers, MA). The following day the membranes were washed four times with TBS‐T, and incubated with Goat anti‐Rabbit IgG HRP‐conjugated secondary antibody (1:5,000; catalog number 170‐6515 Bio‐Rad) for 1 h and then washed four times with TBS‐T. The bands for arginase I, arginase II, EGFR, or pY845EGFR were visualized using chemiluminescence (EMD Millipore, Billerica, MA) and quantified using densitometry (VisionWorks LS, UVP LLC, Upland, CA). To control for protein loading, the blots were stripped and reprobed for *β*‐actin using a monoclonal antibody (1:5,000; catalog number ab6276 Abcam).

### Proliferation assays

To determine cell proliferation, 5000 cells were plated into each well of 6‐well plates. The appropriate treatments were included in the media and the cells were placed in either hypoxia or normoxia for 48 h. At the end of the experimental protocol, the cells were removed from the incubator and plates were washed three times with PBS. After the final wash, 1 mL of trypsin was added to each well. The plates were incubated for ~ 3 min followed by the addition of 2 mL trypsin neutralizing solution. The cells from each well were placed in 15 mL conical tubes. The cells were centrifuged for 5 min at 1220*g* at 4°C. The supernatant was discarded and the cells were resuspended in 1 mL of DMEM. The cells were mixed 1:1 with trypan blue and viable cells were counted using a hemocytometer.

### Statistical analysis

Data are presented as mean ± SE. When only two groups were compared a *t*‐test was used. When more than two groups were compared a one‐way ANOVA was used to compare the data between groups. Differences were identified using a Student–Newman–Keuls posthoc test. All statistics were performed using SigmaPlot (Jandel Scientific, Carlsbad, CA). Differences were considered significant when *P* < 0.05.

## Results

### Hypoxia increases arginase II expression in HeLa cells

**Figure 1:**
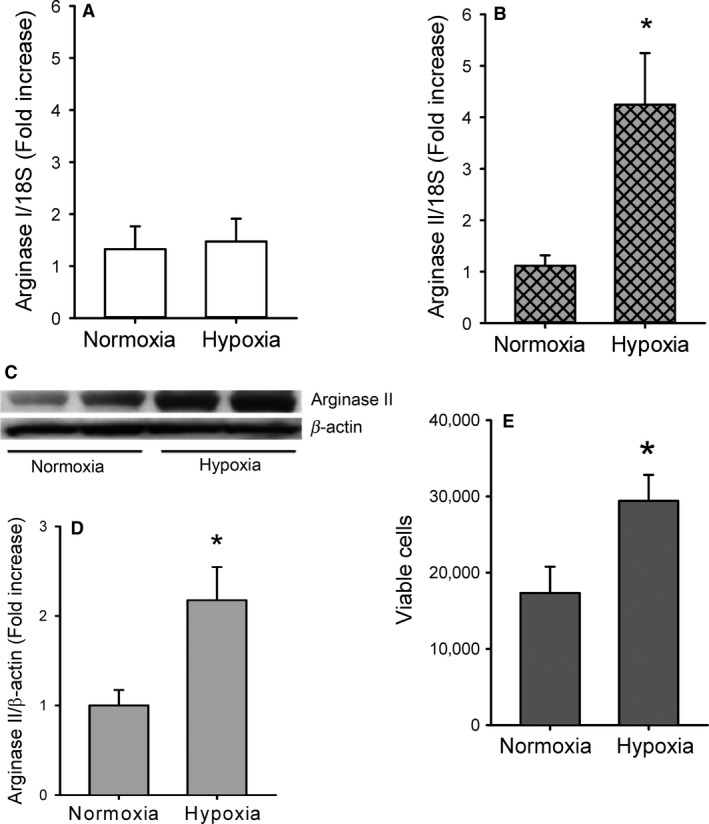
Hypoxia increases arginase II expression and proliferation in HeLa cells. (A) Hypoxia had no apparent effect on arginase I mRNA levels in HeLa cells. Real‐time PCR for arginase I normalized to 18S rRNA levels in HeLa cells exposed to either normoxia (*n* = 6) or hypoxia (*n* = 6) for 24 h. (B) Hypoxia resulted in fourfold greater arginase II mRNA levels in HeLa cells. Real‐time PCR for arginase II normalized to 18S rRNA levels in HeLa cells exposed to either normoxia (*n* = 6) or hypoxia (*n* = 6) for 24 h. *hypoxia different from normoxia, *P* < 0.05. (C) Representative western blot for arginase II from normoxic and hypoxic HeLa cells. (D) Hypoxia resulted in a ~2‐fold increase in arginase II protein levels in HeLa cells (*n* = 5 in each group). Densitometry data from arginase II normalized to *β*‐actin. * hypoxia different from normoxia, *P* < 0.01. (E) Hypoxia increased viable cell numbers in HeLa cells. Equal numbers of HeLa cells were plated in each well of a 6‐well plate and incubated in normoxia (*n* = 9) or hypoxia (*n* = 7) for 48 h. * different from normoxia, *P* < 0.05.

HeLa cells were grown to approximately 90% confluence and incubated in either normoxia or hypoxia for 24 h, to determine the effects of hypoxia on arginase expression. RNA was harvested for determination of arginase I and arginase II mRNA levels by real‐time PCR. Although there was no difference in arginase I mRNA levels between normoxic and hypoxic exposed cells (Fig. 1A), arginase II mRNA expression was ~4‐fold greater in HeLa cells exposed to hypoxia than in those exposed to normoxia for 24 h (Fig. 1B). Lysate protein was harvested and levels of arginase I and arginase II protein in cells exposed to normoxia and hypoxia were determined using Western blot analysis. Consistent with the mRNA data, there was no difference in the protein levels of arginase I in HeLa cells exposed to normoxia or hypoxia for 24 h (data not shown). However, there was ~2‐fold greater arginase II protein levels in the HeLa cells exposed to hypoxia than in those exposed to normoxia for 24 h (Fig. 1D).

### Hypoxia increases proliferation of HeLa cells

Proliferation of HeLa cells exposed to either normoxia or hypoxia was determined by counting viable cells using trypan blue exclusion. A total of 5000 cells were seeded per well on a 6‐well plate and incubated in either normoxia or hypoxia for 48 h. After incubation, viable cell numbers were ~2‐fold greater in hypoxia cells than in normoxic cells (Fig. 1E).

### Arginase inhibition prevents hypoxia‐induced proliferation

**Figure 2:**
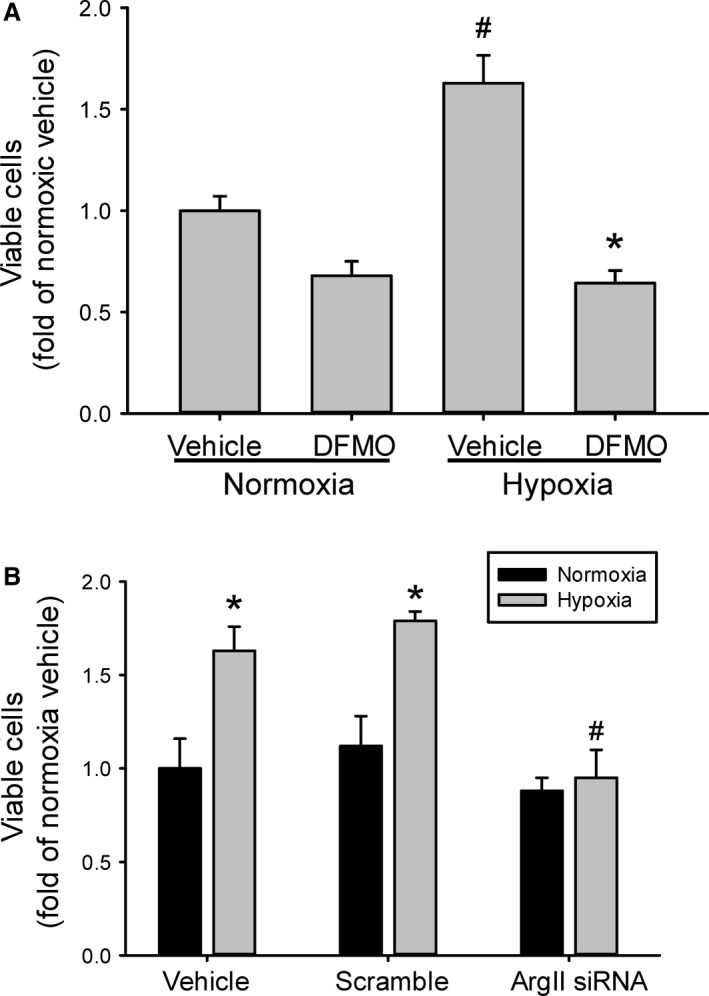
Inhibition of arginase prevented hypoxia‐induced proliferation. (A) Equal numbers of HeLa cells were plated in each well of a 6‐well plate and either vehicle or 1 mmol/L *α*‐difluoromethylornithine (DFMO) was added to the media. The cells were incubated for 48 h in either normoxia (*n* = 3 for each treatment) or hypoxia (*n* = 5 vehicle; *n* = 3 DFMO). # hypoxia different from normoxia, *P* < 0.005. *DFMO different from vehicle same exposure, *P* < 0.001. (B) Cells were either untreated, treated with scramble siRNA, or treated with siRNA against arginase II, after 24 h cells were washed and allowed to recover for 24 h. The cells were then trypsinized and equal numbers of cells plated in each well of a 6‐well plate. After 48 h viable cells were counted using trypan blue exclusion (*n* = 3–7 per group). * hypoxia different from normoxia same treatment, *P* < 0.01. # Hypoxic ArgII siRNA different from hypoxic scramble siRNA,* P* < 0.05.

To determine the role of arginase in hypoxia‐induced HeLa cell proliferation, a nonspecific pharmacological arginase inhibitor, difluoromethylornithine (DFMO; Sigma, St Louis, MO), was used as previously described (Setty et al. 2016). DFMO was added to achieve a concentration of 1 mmol/L and the cells were incubated in either normoxia or hypoxia for 48 h. The viable cell numbers were determined. DFMO had no significant effect on proliferation in the HeLa cells incubated in normoxia (Fig. 2A). Hypoxia incubation significantly increased viable cell numbers after 48 h, and the hypoxia‐induced increase in viable cells was prevented by DFMO (Fig. 2A).

To further examine the specific role of arginase II in hypoxia‐induced HeLa cell proliferation, siRNA against arginase II were used. HeLa cells were either not transfected (vehicle), transfected with scramble siRNA (scramble) or transfected with siRNA against arginase II (ArgII siRNA). After 24 h, the cells were washed and fresh media placed on them for a 24‐h recovery period. Equal numbers of viable cells were seeded in each well of 6‐well plates and placed in either normoxia or hypoxia for 48 h. Viable cell numbers were determined using trypan blue exclusion. Scramble siRNA‐transfected HeLa cells had viable cell numbers that were not different from the nontransfected cells either in hypoxia or normoxia (Fig. 2B). Treatment with arginase II siRNA completely prevented the hypoxia‐induced increase in viable HeLa cells, such that viable cell numbers in the hypoxic arginase II siRNA‐transfected cells were not different from normoxic cells in any of the three treatment groups (Fig. 2B).

### Overexpression of arginase II increased viable cell numbers

**Figure 3:**
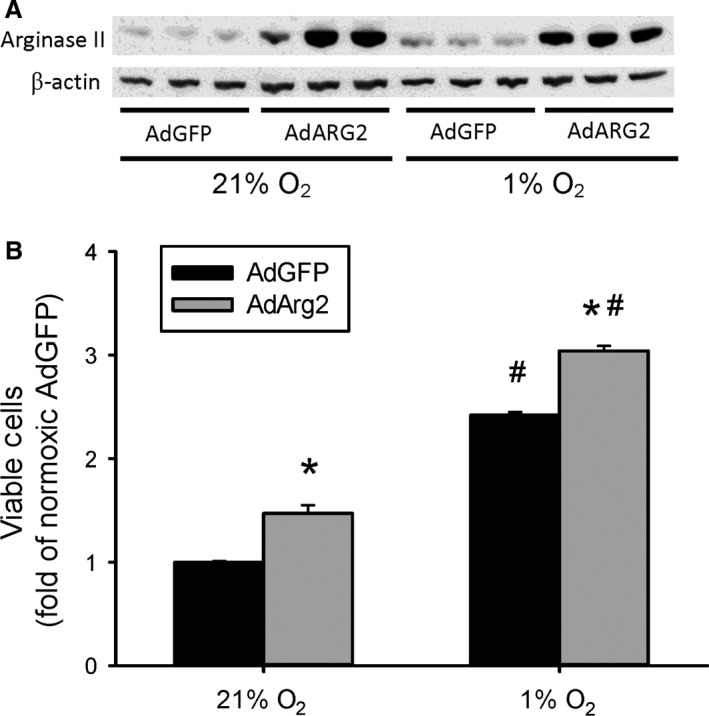
Overexpression of arginase II increased viable cell numbers. HeLa cells were transfected with an adenoviral vector containing either the human arginase II gene (AdArg2) or the gene for green fluorescent protein (AdGFP). (A) Transfection substantially increased arginase II protein levels. Representative western blot for arginase II from cells transfected with either AdGFP or AdArg2 and incubated in either normoxia or hypoxia for 24 h. (B) Cells were transfected with either AdGFP or AdArg2 and 24 h later the cells were washed and fresh media placed on them for a 24 h recovery period. The cells were then trypsinized and equal numbers of cells plated in each well of a 6‐well plate. The cells were then exposed to either normoxia or hypoxia for 48 h and viable cells counted using trypan blue exclusion (*n* = 3 for each group). * AdArg2 different from AdGFP same exposure, *P* < 0.001. # hypoxia different from normoxia same treatment, *P* < 0.001.

To determine the effect of overexpressing arginase II on viable cell numbers, HeLa cells were transfected with an adenoviral vector expressing either the human arginase II gene (AdArg2) or the gene for green fluorescent protein (AdGFP) as a control. Briefly to generate the AdArg2, first the human Arg2 cDNA clone, which contains the open reading frame and a long 3′‐untranslated region, was purchased from Open Biosystems (Huntsville, AL). Second, the AdArg2 and the control AdGFP under the control of a CMV promoter were constructed using the AdEasy Adenoviral Vector System per manufacturer's instructions (Agilent Technologies, La Jolla, CA). Viral stocks had titers ranging from 1 to 5 × 10^10^ plaque‐forming units per mL (pfu/mL) and were stored at −80°C. hPMVEC were tranfected with 100 MOI AdArg2 or AdGFP and after 24 h cells were washed and fresh media placed on them for a 24‐h recovery period. Cells were then trypsinized and equal numbers of cells plated in each well of a 6‐well plate. The cells were then exposed to either normoxia or hypoxia for 48 h and viable cells counted using trypan blue exclusion. Transfection of AdArg2 resulted in greater numbers of viable cells both in normoxia and hypoxia than in the AdGFP‐transfected HeLa cells (Fig. 3).

### EGFR is induced by hypoxia and inhibition of EGFR prevents both hypoxia‐induced arginase II expression and increase in viable cell numbers

**Figure 4:**
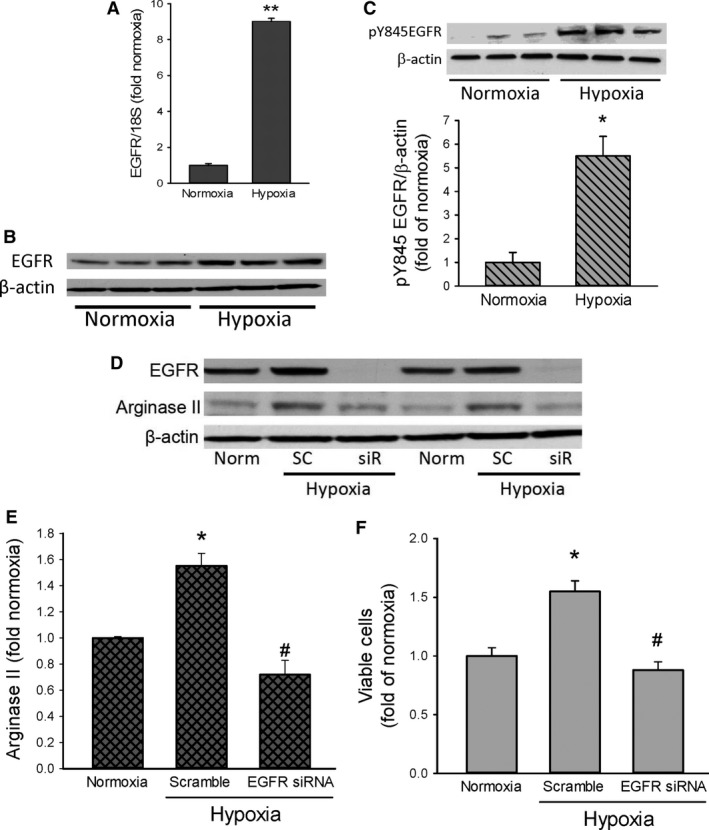
Hypoxia increases EGFR expression and inhibiting EGFR expression prevents hypoxia‐induced arginase II expression and proliferation. (A) EGFR mRNA was greater in hypoxic HeLa cells (*n* = 3) than in normoxic cells (*n* = 3). ** hypoxia different from normoxia, *P* < 0.0001. (B) Representative western blot showing greater EGFR protein levels in hypoxic HeLa cells than in normoxic HeLa cells. (C) Phosphorylated EGFR at tyrosine 845 (pY845EGFR) was greater in hypoxia than in normoxia. Representative western showing hypoxic phosphorylation of tyrosine 845 in EGFR (pY845EGFR). Densitometry data for pY845EGFR normalized to *β*‐actin and expressed as fold of normoxia (*n* = 3 for each exposure). * hypoxia different from normoxia, *P* < 0.01. (D) EGFR siRNA prevented hypoxic EGFR expression. Representative western blot showing that scramble siRNA (SC) had little effect on hypoxic EGFR expression, while the EGFR siRNA (SiR) prevented EGFR expression. (E) The hypoxia‐induced increase in arginase II protein levels was prevented by treatment with an EGFR siRNA. HeLa cells (*n* = 3 in each group) were either untreated, treated with scramble siRNA, or treated with siRNA against EGFR, after 24 h cells were washed and allowed to recover for 24 h. The cells were placed in either normoxia or hypoxia for 48 h, and protein harvested for western blotting for arginase II. * hypoxia different from normoxia, *P* < 0.005. # EGFR siRNA different from scramble siRNA,* P* < 0.002. (F) The hypoxia‐induced increase in viable cell numbers was prevented by the EGFR siRNA. Cells (*n* = 3 for each group) were either untreated, treated with scramble siRNA, or treated with siRNA against EGFR, after 24 h cells were washed and allowed to recover for 24 h. The cells were then trypanized and equal numbers of cells plated in each well of a 6 well plate. After 48 h viable cells were counted using trypan blue exclusion. * hypoxia different from normoxia, *P* < 0.005. # EGFR siRNA different from scramble siRNA,* P* < 0.005.

To determine the effect of hypoxia on the expression of EGFR, HeLa cells were either incubated in normoxia or hypoxia for 24 h and mRNA harvested for qPCR analysis of EGFR. HeLa cells exposed to hypoxia had ~9‐fold greater levels of EGFR mRNA than did cells exposed to normoxia (Fig. 4A). Similarly, after normoxic or hypoxic exposure cell lysate protein was harvested for western blot analysis of EGFR and EGFR phosphorylated at tyrosine 845 (pY845EGFR). Hypoxic HeLa cells had greater protein levels of EGFR protein than did normoxic HeLa cells (Fig. 4B). Furthermore, the hypoxic cells had greater levels of EGFR protein that was phosphorylated at tyrosine 845 (pY845EGFR) than did normoxic cells (Fig. 4C).

We have previously described that in pulmonary endothelial cells hypoxia‐induced proliferation depends on EGFR activation and resultant induction of arginase II expression [5]. To determine if EGFR activation has a role in hypoxia‐induced proliferation of HeLa cells, we used siRNA to knockdown EGFR expression. The HeLa cells were treated with vehicle, scramble siRNA, or EGFR siRNA for 24 h. The hPMVEC were then washed and allowed to recover in normoxia for 24 h. The cells were then placed in either normoxia or hypoxia for 24 h, and cell protein was harvested for western blot analysis. The EGFR siRNA knocked down EGFR expression in hypoxic HeLa cells (Fig. 4D), whereas scramble RNA had no effect on EGFR expression. Treatment with the scramble had little effect on the hypoxia‐induced increase in arginase II protein levels, while treatment with the EGFR siRNA prevented the hypoxia‐induced increase in arginase II protein levels (Fig. 4E). To determine the effect of EGFR siRNA on hypoxia‐induced proliferation in HeLa cells, the cells were treated as above. The cells were then washed, fresh media placed on them for a 24‐h recovery period. An equal number of cells were then seeded into each well of 6‐well plates and placed in either normoxia or hypoxia for 48 h. Viable cell numbers were determined using trypan blue exclusion. The scramble siRNA had little effect on the hypoxia‐induced increase in viable HeLa cell numbers, while the EGFR siRNA prevented the hypoxia‐induced increase in viable HeLa cell numbers (Fig. 4F).

### EGF induces arginase II and increases viable cell number through EGFR in normoxia

**Figure 5:**
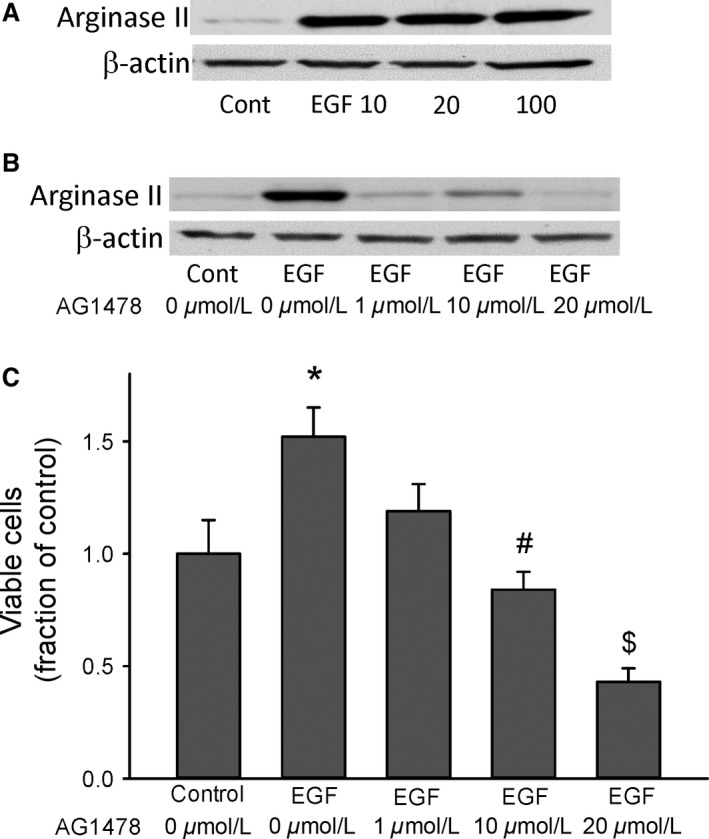
EGF increased arginase II expression and viable cell numbers in HeLa cells. (A) Representative western blot for arginase II, cells were incubated in normoxia for 24 h with either no treatment or EGF (10, 20, or 100 ng) added to the media. Addition of EGF resulted in easily detectable arginase II protein levels in normoxic HeLa cells. (B) The EGF‐induced increase in arginase II was prevented by the small molecule EGFR inhibitor AG1478. Representative western blot for arginase II, cells were incubated in normoxia for 24 h with either no treatment or EGF (10 ng) added to the media and either vehicle (0 *μ*mol/L AG1478) or increasing concentrations of AG1478 (1, 10, or 20 *μ*mol/L). Addition of AG1478 prevented the EGF‐induced increase in arginase II protein levels in normoxic HeLa cells. (C) EGF increased viable cell numbers, an effect that was prevented by treatment with AG1478. Equal numbers of HeLa cells were plated in each well of 6‐well plates. Cells (*n* = 3 in each group) were either untreated or treated with 10 ng EGF, cells were also treated with either vehicle or increasing concentrations of AG1478. After incubating in normoxia for 48 h viable cells were counted using trypan blue exclusion. * EGF different from control, *P* < 0.05. # 10 *μ*mol/L AG1478 treatment different from EGF alone, *P* < 0.01. $ 20 *μ*mol/L AG1478 different from EGF alone, *P* < 0.001.

To determine the effect of EGF on arginase II protein levels, HeLa cells were incubated in normoxia for 24 h with either no treatment or EGF (10, 20, or 100 ng/mL) added to the media. Addition of EGF resulted in substantial arginase II protein levels in normoxic HeLa cells (Fig. 5A). To determine the role of EGFR activation by EGF in the EGF‐mediated increase in arginase II protein levels we used the putative EGFR inhibitor AG1478. HeLa cells were treated with either vehicle (control) or 20 ng/mL of EGF and either vehicle (0 *μ*mol/L) or increasing concentrations of AG1478 (1, 10, or 20 *μ*mol/L) were added to the media. The cells were then incubated for 24 h in normoxia and protein harvested for arginase II western blotting. EGF‐induced arginase II protein expression and AG1478 prevented the EGF‐induced increase in arginase II expression (Fig. 5B).

To determine the effect of EGF and EGF‐mediated activation of EGFR on viable cell numbers we again used AG1478. An equal number of cells were seeded into each well of 6 well plates. The cells were treated with either vehicle (control) or 20 ng/mL of EGF and either vehicle (0 *μ*mol/L) or increasing concentrations of AG1478 (1, 10, or 20 *μ*mol/L) were added to the media.

The cells were then placed in normoxia for 48 h. Viable cell numbers were determined using trypan blue exclusion. EGF resulted in greater numbers of viable cells, and the EGF‐induced increase in viable cell numbers was prevented in a concentration‐dependent manner by AG1478 (Fig. 5C).

### Hypoxia induces EGF expression and blocking EGF prevents hypoxia‐induced proliferation

**Figure 6:**
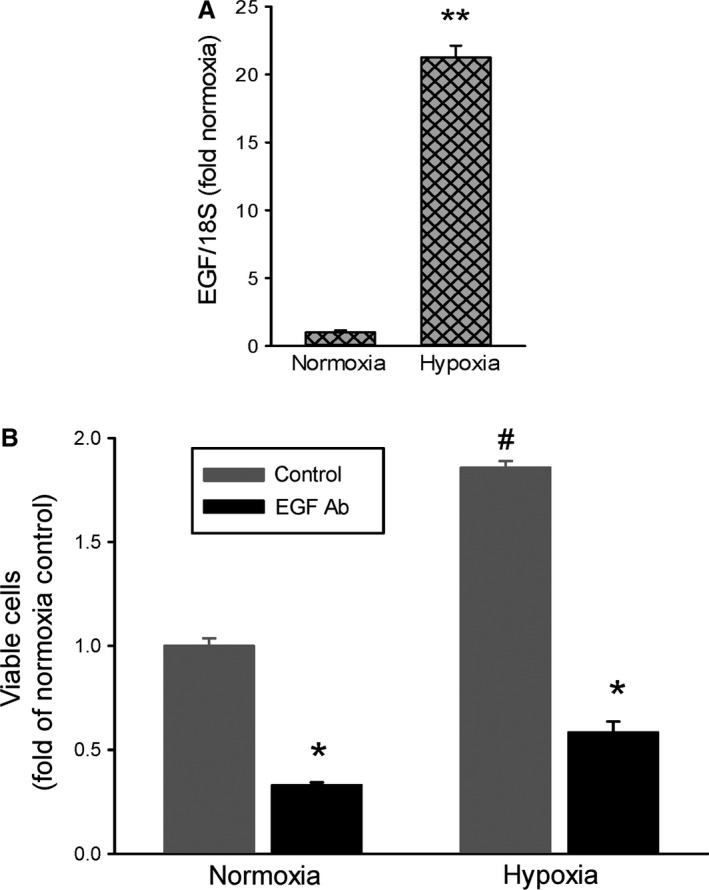
EGF neutralizing antibody (EGF Ab) prevented the hypoxia‐induced increase in viable cell numbers. (A) EGF mRNA was substantially greater in hypoxic HeLa cells than in normoxic cells (*n* = 3 in each exposure). ** hypoxia different from normoxia, *P* < 0.0001. (B) Neutralizing EGF prevented the hypoxia‐induced increase in viable cell numbers. Equal numbers of HeLa cells were plated in each well of a 6‐well plate. Cells (*n* = 3 in each group) were treated with 3 *μ*g of either IgG or EGF Ab and placed in either normoxia or hypoxia for 48 h. Viable cells were counted using trypan blue exclusion. * EGF Ab different from control in same exposure, *P* < 0.001. # hypoxia different from normoxia, *P* < 0.001.

To determine the effect of hypoxia on the expression of EGF, HeLa cells were either incubated in normoxia or hypoxia for 24 h and mRNA harvested for qPCR analysis of EGF. HeLa cells exposed to hypoxia had ~20‐fold greater levels of EGF mRNA than did cells exposed to normoxia (Fig. 6A).

To determine the effect of preventing EGF interactions on the hypoxia‐induced increase in viable cell numbers we utilized an antibody that binds EGF and prevents its interaction with surface receptors, which we refer to as an EGF neutralizing antibody. An equal number of cells were seeded into each well of 6‐well plates. The cells were treated with either isotype control IgG (control) or an EGF neutralizing antibody. The cells were placed in either normoxia or hypoxia for 48 h and viable cell numbers were determined using trypan blue exclusion. Hypoxia resulted in greater viable cell numbers than did normoxia (Fig. 6B). The hypoxia‐induced increase in viable cells was prevented by the EGF neutralizing antibody (Fig. 6B). It should be noted that treatment with the EGF neutralizing antibody also resulted in fewer viable cells in normoxia (Fig. 6B).

### Blocking ligand‐binding to EGFR prevents both EGF‐induced and hypoxia‐induced proliferation

**Figure 7:**
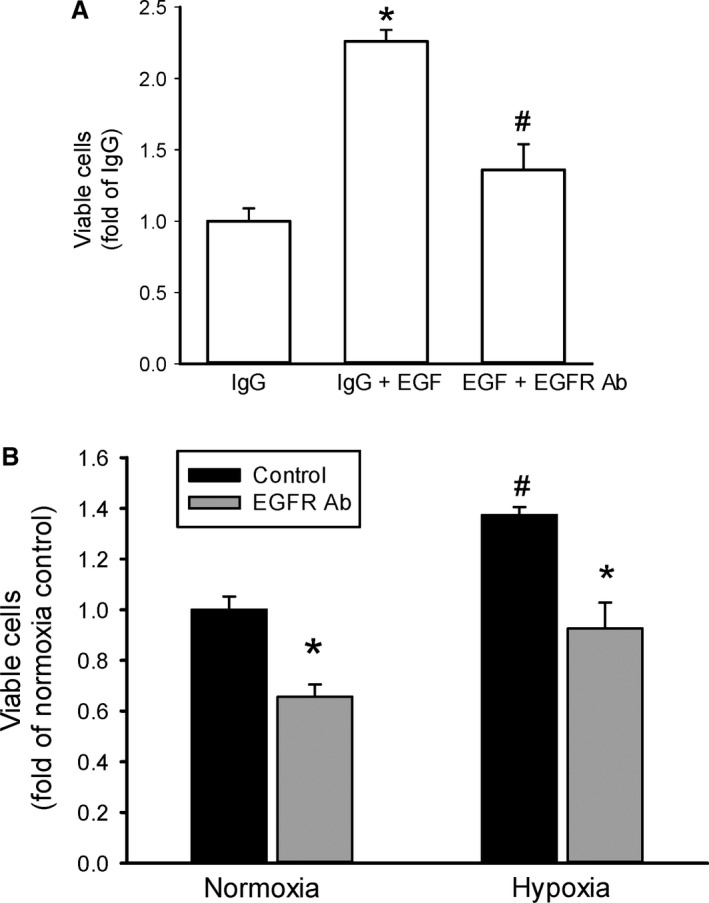
Inhibiting EGFR activation using an EGFR blocking antibody (EGFR Ab) prevented the hypoxia‐induced increase in viable cell numbers. A) EGFR Ab prevented the EGF‐induced increase in viable cell numbers. Equal numbers of HeLa cells were plated in each well of 6‐well plates. Cells (*n* = 3 in each group) were either untreated or treated with 10 ng EGF, and cells were also treated with 3 *μ*g of either IgG or EGFR Ab. After 48 h in normoxia viable cell numbers were counted using trypan blue exclusion. * IgG different from IgG + EGF,* P* < 0.005. # EGF + EGFR Ab different from EGF+ IgG, *P* < 0.005. (B) EGFR Ab prevented the hypoxia‐induced increase in viable cell numbers. Equal numbers of HeLa cells were plated in each well of a 6‐well plate. Cells (*n* = 3 in each group) were treated with 3 *μ*g of either IgG (control) or EGFR Ab and exposed to either normoxia or hypoxia for 48 h. Viable cells were counted using trypan blue exclusion. * EGFR Ab different from control same exposure, *P* < 0.05. #hypoxia different from normoxia same treatment, *P* < 0.005.

To determine the effect of blocking ligand interaction with EGFR on viable cell numbers we utilized an antibody that binds EGFR so that ligand cannot bind. An equal number of cells were seeded into each well of 6 well plates. The cells were treated with either vehicle or 20 ng/mL of EGF and either IgG or the EGFR blocking antibody. The cells were then placed in normoxia for 48 h. Viable cell numbers were determined using trypan blue exclusion. In cells treated with isotype control IgG, EGF resulted in substantially greater numbers of viable cells than did vehicle (Fig. 7A). The EGF‐mediated increase in viable cell numbers was prevented by the EGFR blocking antibody (Fig. 7A).

Similarly, the EGFR blocking antibody was used to determine the effect of blocking ligand interactions with EGFR on the hypoxia‐induced increase in viable cell numbers. An equal number of cells were seeded into each well of 6‐well plates. The cells were treated with either IgG (control) or the EGFR blocking antibody. The cells were placed in either normoxia or hypoxia for 48 h and viable cell numbers were determined. Hypoxia resulted in greater viable cell numbers than did normoxia (Fig. 7B). The hypoxia‐induced increase in viable cells was prevented by the EGFR blocking antibody (Fig. 7B), but not by the isotype control antibody. Again it should be noted that treatment with the EGFR blocking antibody also resulted in fewer viable HeLa cells in normoxia (Fig. 7B).

## Discussion

The aim of this study was to determine the effects of hypoxia on arginase expression and cell proliferation and delineate the underlining signal transduction pathways involved. The main findings of this study were that in HeLa cells: (1) hypoxia increased arginase II mRNA and protein expression; (2) hypoxia‐induced cell proliferation was dependent on arginase II induction; (3) hypoxia‐induced EGFR expression and inhibiting EGFR activity prevented arginase II induction and proliferation; (4) EGF‐induced arginase II expression and cell proliferation through an EGFR‐mediated pathway; and (5) hypoxia‐induced EGF expression and inhibiting EGF‐EGFR interaction prevented hypoxia‐induced cell proliferation. Taken together, these findings support our hypotheses, that hypoxia results in HeLa cell proliferation due to arginase induction, and that the hypoxia‐induced arginase II induction and proliferation depends on EGF‐mediated activation of EGFR.

To our knowledge, the effects of hypoxia on arginase expression in human cervical cancer cells have not been shown before, although arginase activity in HeLa cells was described in 1983 (Pohjanpelto and Hölttä 1983). Furthermore, a central role for arginine, the substrate for arginase, in the multi‐step processes resulting in cancer have been described (Wheatley et al. 2003). An essential role for arginine has been shown for multiple cancer cell lines, where depriving cancer cells of arginine, the substrate for cellular arginase, and thus analogous to studies like ours using arginase inhibitors, resulted in cell death (Scott et al. 2000). We found that HeLa cells expressed arginase I but that hypoxia did not result in significant changes in arginase I protein levels. On the other hand, arginase II gene and protein expression were both upregulated in response to hypoxia. These data suggest that it is arginase II that is the hypoxia‐inducible isoform in HeLa cells. This observation is consistent with our previous findings in osteosarcoma cells (Setty et al. 2016), human pulmonary microvascular endothelial cells (Toby et al. 2010), and human pulmonary arterial smooth muscle cells (Chen et al. 2009). Hypoxia has also been shown to increase arginase expression in a variety of other cells. An increase in arginase activity was shown in the brain of rats exposed to a hypoxic insult (Clarkson et al. 2005). Similar findings were described in rat wound‐derived and mouse peritoneal macrophages in both hypoxic and anoxic culture (Louis et al. 1998). Our results demonstrate the central role of arginase II in hypoxia‐induced HeLa cell proliferation. This is also consistent with our recent findings in osteosarcoma cell lines, wherein inhibition of arginase activity using small molecule inhibitors prevented hypoxia‐induced increases in viable cell numbers, while preventing arginase II induction using siRNA also prevented the hypoxia‐induced increase in viable cell numbers (Setty et al. 2016). Hypoxia is a characteristic feature of locally advanced solid tumors. Neoplastic cells under hypoxic conditions tend to promote tumor cell growth rather than apoptosis, and can influence a more aggressive phenotype that is associated with a poorer prognosis (Vaupel and Mayer 2007). Thus, our finding of an important role of arginase II in hypoxia‐induced proliferation of HeLa cells suggests a novel therapeutic target that could potentially target tumor cell growth. Although outside the scope of this study, arginase has been shown in a variety of conditions to be a regulator of cellular nitric oxide production by nitric oxide synthase (Li et al. 2001; Durante et al. 2007), thus future studies examining the role of arginase on NO and peroxynitrite production, and related effects on cell viability in hypoxic HeLa cells would be important.

EGFR induction by hypoxia has been shown in many types of cells including many types of cancer cells (Nishi et al. 2002; Swinson and O'Byrne 2006; Franovic et al. 2007; Wouters et al. 2013). Indeed preclinical and clinical studies support a role for hypoxia‐induced EGFR upregulation in cancers without genetic mutations of EGFR (Wang et al. 2007). Thus, it is not surprising that hypoxia also induced EGFR expression in HeLa cells. From this stand‐point, it is worth‐noting that several studies have evaluated the efficacy of combinational therapy consisting of EGFR inhibitors and radiation in advanced cervical cancers (Herrera et al. 2008). In breast cancer overexpression of EGFR correlates with poor prognosis, and EGFR targeting antibodies and EGFR receptor tyrosine kinase inhibitors are used to treat advanced breast cancers (Ciardiello and Tortora 2008). Previously it was reported that in HeLa cells that the early growth response‐1 (Egr‐1) gene mediates upregulation of EGFR expression in hypoxia (Nishi et al. 2002). In other cancer cell lines it was reported that hypoxia increased EGFR expression and this was dependent on hypoxia‐inducible factor 1‐*α* activation (Swinson and O'Byrne 2006). The exact mechanism that leads to EGFR expression is outside the scope of the current work. However, EGFR has been shown in many cancer types to confer a survival advantage, that is to say it is pro‐proliferative and anti‐apoptotic. Our findings suggest that one potential mechanism of the survival advantage in HeLa cells of hypoxia‐induced EGFR is by the upregulation of arginase II and the resulting increase in viable cell numbers.

EGFR can be activated by ligand‐binding, and ligands include epidermal growth factor (EGF), epidermal growth factor‐like molecules, neuroregulins, and transforming growth factor‐*α* (TGF‐*α*). Our results demonstrate that EGF is potently upregulated by hypoxia. Such upregulation of EGF expression is likely to be biologically significant in arginase II induction and cell proliferation, since EGF treatment also upregulated arginase II and increased viable cell numbers in HeLa cells. Finally, we found that when EGF‐binding to EGFR was blocked the hypoxia‐induced increase in viable cell numbers was prevented. Furthermore, the finding that the EGF neutralizing antibody prevented the hypoxia‐induced increase in viable cell numbers supports the notion that hypoxia‐induced EGF production serves as an autocrine loop to activate EGFR in cancers. Our results using the EGFR blocking antibody demonstrate that EGF must bind to EGFR to exert pro‐proliferative effects. Finally, our data is consistent with the idea that hypoxia‐induced EGF/EGFR signaling mediates the proliferative response through the induction of arginase II expression and activity. Thus, we propose an EGF/EGFR/arginase II pathway leading to the hypoxic proliferation seen in HeLa cells. Our model suggests that the EGF/EGFR/arginase II pathway represents a potential therapeutic target to prevent hypoxia induced proliferation in tumor cells. Furthermore, hypoxia can lead to increased metastasis, at least in certain tumor cell types (Vaupel and Mayer 2007), thus inhibiting the EGF/EGFR/arginase II pathway may also potentially have an additional benefit in limiting metastasis.

## Conflict of Interest

None declared.
